# Novel Cinnamic Acid Derivatives as Antioxidant and Anticancer Agents: Design, Synthesis and Modeling Studies

**DOI:** 10.3390/molecules19079655

**Published:** 2014-07-07

**Authors:** Eleni Pontiki, Dimitra Hadjipavlou-Litina, Konstantinos Litinas, George Geromichalos

**Affiliations:** 1 Department of Pharmaceutical Chemistry, School of Pharmacy, Faculty of Health Sciences, Aristotle University of Thessaloniki, Thessaloniki 54124, Greece; 2 Laboratory of Organic Chemistry, Department of Chemistry, Aristotle University of Thessaloniki, Thessaloniki 54124, Greece; 3 Symeonidion Research Center, “Theagenion” Cancer Hospital, Thessaloniki, 54007, Greece

**Keywords:** antioxidant activity, inflammation, cancer, lipoxygenase, cinnamic acids, molecular docking

## Abstract

Cinnamic acids have been identified as interesting compounds with antioxidant, anti-inflammatory and cytotoxic properties. In the present study, simple cinnamic acids were synthesized by Knoevenagel condensation reactions and evaluated for the above biological activities. Compound **4ii** proved to be the most potent LOX inhibitor. Phenyl- substituted acids showed better inhibitory activity against soybean LOX, and it must be noted that compounds **4i** and **3i** with higher lipophilicity values resulted less active than compounds **2i** and **1i**. The compounds have shown very good activity in different antioxidant assays. The antitumor properties of these derivatives have been assessed by their 1/IC_50_ inhibitory values in the proliferation of HT-29, A-549, OAW-42, MDA-MB-231, HeLa and MRC-5 normal cell lines. The compounds presented low antitumor activity considering the IC_50_ values attained for the cell lines, with the exception of compound **4ii**. Molecular docking studies were carried out on cinnamic acid derivative **4ii** and were found to be in accordance with our experimental biological results.

## 1. Introduction

Cancer has become the second major cause of death in developed countries and the clinical prognosis remains relatively poor. During the last decade extensive research has been done in order to define the mechanism by which continued oxidative stress could lead to cancer and chronic inflammation.

Although inflammation has long been recognized as a risk and causative factor for several types of cancer, many of the molecular and cellular mechanisms involved still remain unclear. The functional relationship between polyunsaturated fatty acid metabolism, inflammation, and carcinogenesis has been extensively examined in numerous molecular studies, revealing potential novel targets like arachidonic acid metabolizing enzymes, such as cyclooxygenases (COXs) and lipoxygenases (LOXs) [1,2]. The formation by these enzymes of various lipid peroxides and bioactive lipids, free radicals, and aldehydes can induce deleterious gene mutation and post-translational modifications of key cancer-related proteins, implicated in the regulation of cellular proliferation, apoptosis, differentiation, and senescence [3,4,5]. Especially lipoxygenase-catalyzed products may exert their biological effects in an intracrine manner, through the activation of transcription factors of the peroxisome proliferator-activated receptor/PPAR family, or may interact with specific trans-membrane G protein-coupled cell surface receptors in an autocrine or paracrine manner [6,7].

High levels of ROS are able to modify essentially biological molecules, such lipids, proteins and DNA. It is consistent that rates of ROS production are increased in most diseases [5]. Under “oxidative stress” conditions, reactive oxygen species (ROS) in the form of superoxide anion, hydroxyl radical and hydrogen peroxide attack various biological macromolecules (proteins, enzymes, DNA, *etc.*) or indirectly may interfere with mechanisms of DNA repair [8]. Oxidative stress can activate signal transduction pathways, inducing changes in a variety of transcription factors (nuclear factor/NF-κB, activator proteion/AP-1, hypoxia-inducible factor-1α/HIF-1^α^, nuclear factor of activated T-cells and NF-E2 related factor-2/Nrf2) leading to the expression of genes, including those for growth factors, inflammatory cytokines, chemokines, cell cycle regulatory molecules, and anti-inflammatory molecules that may induce a high perturbation in the intracellular and intercellular homeostasis, genetic instability, proliferation, chemoresistance, radioresistance, invasion, angiogenesis, and stem cell survival [9].

It is of increasing interest that the treatment of the above-mentioned pathophysiological conditions could benefit from the use of drugs that combine the above activities. The urgency and the growing need for multifunction molecules designed to block multiple targets in cancer cells are well accepted.

During the last decade, natural products bearing the cinnamoyl moiety have attached much attention due to their broad spectrum of biological activities and low toxicity. Additionally, *trans-*cinnamic acid derivatives, both isolated from plant sources or synthesized, are well known for their antioxidant [10], antitumor [11], antimicrobial [12] and antimycobacterial properties [13]. Cinnamic acid derivatives, especially those combining the cinnamoyl moiety with hydroxyl groups, present strong free radical scavenging properties. Acids, esters, amides, hydrazides and related derivatives of cinnamic acid with such activities are reported in the literature for their health benefits [14,15]. Especially, *p*-coumaric acid or 4-hydroxy-*trans*-cinnamic acid presents antioxidant activity, involving direct scavenger of reactive oxygen species (ROS) by minimizing the oxidation of low-density lipoprotein (LDL) [16]. Lipophilic hexylamides and hexylesters of cinnamic and hydrocinnamic acids as well as the corresponding acid precursors, have been recently studied for their antioxidant profile and found to play important role in neurodegenerative diseases (ND) due to their ability to cross the blood-brain barrier [17]. Additionally, 2'-hydroxycinnamaldehyde and the analogue 2'-benzoyloxycinnamaldehyde induce apoptosis in cancer cells *via* the induction of cellular reactive oxygen species (ROS) [18]. Recently antitumor activities of various cinnamic acid derivatives were explored by many research groups [19,20,21,22].

In this study we have chosen to synthesize a series of substituted cinnamic acids based on the fact that the cinnamoyl moiety has been found in a variety of biologically active substances, as already mentioned. Previous studies of our group have shown that cytotoxic and antiinflammatory effects of antioxidant cinnamic acids are associated with their pro-oxidant effects [23,24,25,26]. The antioxidant, anti-inflammatory and anticancer properties of cinnamic acids are known to be influenced to a great extent by the substitutions of the aryl ring and the double bond. In order to enhance the anticancer and anti-inflammatory activity of these derivatives [25] a series of new cinnamic acids with the appropriate substituents have been synthesized. These series have been evaluated for their: (a) antioxidant activity in different assays (b) anticancer activity in different cell lines and (c) ability to inhibit soybean lipoxygenase. Judging the *in vitro* results representative derivatives are further subjected to modeling studies.

## 2. Results and Discussion

### 2.1. Chemistry

**Scheme 1:**
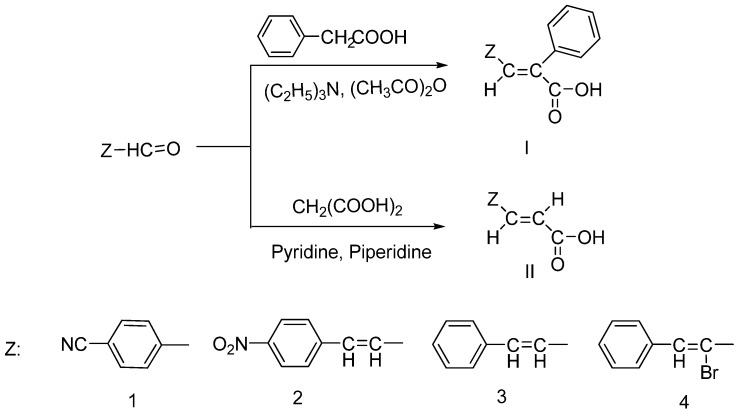
Synthesis of cinnamic acids.

The synthesis of cinnamic acids was accomplished by a Knoevenagel condensation reaction as already reported by us [24] and shown in Scheme 1. Acids of series **i** were derived from the condensation of the suitable cinnamic aldehyde or not with 3-phenylacetic acid and acetic acid anhydride in the presence of triethylamine, while 3-substituted-acrylic acids of series **ii**, were derived from the condensation of the suitable aldehydes with malonic acid in the presence of pyridine and piperidine.

Products **1i**, **1ii**, **2i**, **2ii** were obtained in satisfactory yields (55%–77%), **4i** in 33% yield and products **3i**, **3ii** and **4ii** only in traces (Table 1). The pure final products were recrystallized from ethanol/water. IR, ^1^H-NMR, ^13^C-NMR and elemental analysis were used for the confirmation of the synthesized compounds structures (Table 1).

**Table 1:** Chemical structures, physicochemical and reaction data of cinnamic acid derivatives **1**–**4.** 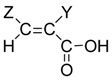

No	Z	Y	Formula *	R_f_	Clog *P* ^**^	R_M_^#^ (±SD)	Mp °C	Yield%
**1i**	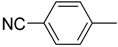	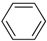	C_16_H_11_NO_2_	0.74 ^a^	3.20	−0.895 (0.004) ^e^	155–157	60
**1ii**	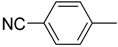	H	C_10_H_7_NO_2_	0.48 ^a^	1.61	−0.972 (0.032) ^e^	258–260	67
**2i**	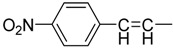	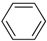	C_17_H_13_NO_4_	0.39 ^b^	3.58	−0.706 (0.062) ^e^	163–165	77
**2ii**	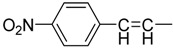	H	C_11_H_9_NO_4_	0.52 ^b^	2.00	−0.868 (0.019) ^e^	170–172	55
**3i**	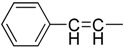	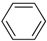	C_17_H_14_O_2_	0.88 ^b^	3.84	0.987 (0.018) ^e^	145–147	12
**3ii**	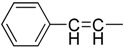	H	C_11_H_10_O_2_	0.62 ^b^	2.25	−0.893 (0.002) ^e^	163–165	9
**4i**	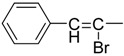	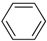	C_17_H_13_BrO_2_	0.85 ^c^	4.09	0.566 (0.001) ^e^	135–136	33
**4ii**	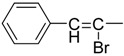	H	C_11_H_9_BrO_2_	0.89 ^d^	2.51	−0.847 (0.044) ^e^	169–171	3

^a^ C_6_H_5_:C_2_H_5_OH (1:1); ^b^ CH_3_COOCH_2_CH_3_:CHCl_3_:petroleum ether (2:1:1); ^c^ CHCl_3, _CH_2_Cl_2_:CH_3_COOC_2_H_5_ (2:1); ^d^ CH_3_COOCH_2_CH_3_:CHCl_3_:petroleum spirit (2:1:1); ^e^ CH_3_OH:H_2_O:CH_3_COOH, (77: 23: 0.1); * Elemental analyses for molecular formula (±0.4%), ** Theoretically calculated clog *P* values; ^#^ R_M_ values are the average of at least 10 measurements.

The syntheses of compounds **1ii** [27], **2ii** [28] and **3ii** [29] have been previously reported. Compound **1i** has been isolated in the *cis-* form by Chapman *et al.* [30] whereas no evidence for the stereochemistry of **3i** has been given [31,32].

### 2.2. Physicochemical Studies

Lipophilicity represents a significant physicochemical property for the absorption, distribution, metabolism and excretion of drugs (ADME properties). In this study the experimentally lipophilicity as R_M_ values is measured by the reverse-phase thin layer chromatography (RPTLC), a reliable, fast and convenient method. The obtained values were compared with the corresponding log *P* values in n-octanol-buffer estimated by Clog*P* [33,34]. It is well known that lipophilicity plays an important role at the ADME properties. In the present study additionally, it influences the radical scavenging property (*i.e*., in an aqueous phase) or the chain-breaking antioxidant activity (*i.e*., in biological membranes).

An attempt to correlate log *P* and R_M_ values (Table 1) did not succeed. The different nature of the hydrophilic and lipophilic phases used in the two systems is the main reason.

### 2.3. Biological Assays

In this study a series of eight cinnamic acids were synthesized, by application of standard synthetic methods summarized in Scheme 1, expected to offer inhibition of LOX, protection against radical attack and cytotoxicity. Antioxidants acting as lipid peroxidation inhibitors could offer for health’s maintainance and to the compensation of risk factors [5]. Several different antioxidant protocols have been reported and subjected in critical review [35]. They are all available to be used in order to assess *in vitro* antioxidant ability. However, they usually give inconsistent and conflicting responses. Since the antioxidant capacity of a compound must be evaluated in a variety of milieus, factors such solubility or steric hindrance must be considered seriously. Also each assay is related to the generation of a different radical.

The evaluation of the novel acids against soybean lipoxygenase LOX was accomplished by the UV-based enzyme assay of Pontiki and Hadjipavlou-Litina [23,24,25]. This assay may be used as a qualitative or semi-quantitative screen for such activity [36].

Study of LOX IC_50_ inhibition values demonstrates that compound **4ii** is by far the most active inhibitor, followed by compounds **1i** and **2i** (Table 2). It seems that the introduction of a phenyl ring at position 2 favours the inhibitory activity, with the exception of compound **4ii**. The rest of the compounds presented moderate inhibitory activity. In compounds **1i**, **2i**, **3i** and **4i** the presence of a conjugated double bond does not seem to offer to the activity. Thus, compound **1i** is more potent than the conjugated acids **2i**, **3i**, **4i**. Compounds **3i** and **4i** seem to be equipotent. Thus, the replacement of an H-atom by a Br-atom in position α does not influence lipoxygenase inhibition. On the contrary a *p*-NO_2_ substituent offers to the inhibition (**2i** > **3i**). Small differences were observed between **2ii** and **3ii**.

Most of the LOX inhibitors present antioxidant activity or act as free radical scavengers [37] since lipoxygenation occurs via a carbon centered radical. It has been found that LOX inhibition is correlated to the reducing ability of the inhibitors of the Fe^3+^ at the active site to the catalytically inactive Fe^2+^ [38,39]. Several LOX inhibitors proved to be excellent ligants for Fe^3+^. 

Lipophilicity is referred [23,24,25] to as an important physicochemical property for LOX inhibition. However, herein highly lipophilic compounds **4i** and **3i** resulted less active than compounds **1i** and **2i** (Table 2).

**Table 2:** *In vitro* lipoxygenase (LOX) inhibitory activity of cinnamic acid derivatives **1**–**4**. Percent interaction of the cinnamic acids **1**–**4** with the stable radical 1,1-diphenyl-picrylhydrazyl (DPPH).

Compd.	LOX ^a^ IC_50_ (μM) (±SD) ^b^	RA % 0.05 mM (±SD) ^b^		RA % 0.1 mM (±SD) ^b^	
		20 min	60 min	20 min	60 min
**1i**	60 ± 0.4	12 ± 0.3	14 ± 0.1	28 ± 0.6	20 ± 0.8
**1ii**	27% ± 0.8 (0.01 mM)	13 ± 0.3	9 ± 0.03	23 ± 0.5	21 ± 1.0
**2i**	66.5 ± 1.2	10 ± 0.04	11 ± 0.02	23 ± 0.7	23 ± 0.9
**2ii**	31% ± 0.7 (0.01 mM)	11 ± 0.0.2	13 ± 0.04	29 ± 1.0	24 ± 0.6
**3i**	74 ± 1.7	4 ± 0.02	3 ± 0.0	5 ± 0.0	6 ± 0.04
**3ii**	24% ± 0.4 (0.01 mM)	11 ± 0.06	7 ± 0.0	20 ± 0.2	16 ± 0.05
**4i**	74 ± 1.8	7 ± 0.03	4 ± 0.01	8 ± 0.03	5 ± 0.0
**4ii**	10 ± 0.05	17 ± 0.02	13 ± 0.05	30 ± 0.9	29 ± 0.6
**NDGA**	28 ± 0.4	85 ± 1.6	83 ± 1.1	81 ± 0.8	83 ± 0.7

^a^ Soybean lipoxygenase inhibition expressed as IC_50_ (μM); ^b^ Values are means ± SD of three or four different determinations. Means within each column differ significantly (*p* < 0.05).

As already mentioned free radicals are highly implicated in lipoxygenase inhibition, inflammation and cancer. Compounds possessing these activities in combination with antioxidant activity might be protective against these diseases and lead to active and useful drugs, so it would be interesting to evaluate these new acids for their antioxidant activities in comparison to well known antioxidants, *i.e.*, nordihydroguaretic acid (NDGA), Trolox and caffeic acid (Table 2, Table 3 and Table 4).

**Table 3:** Radical scavenging activity of cinnamic acids derivatives **1**–**4** of hydroxyl (HO**^·^** %) and superoxide (O_2_^−**·**^, %) radicals. Antioxidant activities of cinnamic acids **1**–**4** in ABTS^+^ - decolorization (ABTS^+^%) and inhibition of linoleic acid peroxidation (AAPH) assays.

Compd.	HO (%) 0.1 mM (±SD) ^a^	O_2_^−·^·(%) 0.1 mM (±SD) ^a^	ABTS^+^·(%) 0.1 mM (±SD) ^a^	AAPH (%) 0.1 mM (±SD) ^a^
**1i**	no	75 ± 1.0	26 ± 0.4	92 ± 1.2
**1ii**	no	50 ± 0.5	47 ± 0.8	20 ± 0.1
**2i**	33 ± 0.8	no	26 ± 0.5	89 ± 0.9
**2ii**	no	no	35 ± 0.9	29 ± 0.7
**3i**	81 ± 1.9	no	26 ± 0.8	84 ± 1.4
**3ii**	81 ± 1.2	no	31 ± 1.1	30 ± 1.0
**4i**	no	no	24 ± 0.5	88 ± 1.8
**4ii**	100 ± 1.8	no	26 ± 0.4	63 ± 1.1
**Trolox**	73 ± 1.0	-	88 ± 1.7	63 ± 1.3
**Caffeic acid**	-	46 ± 0.5	-	-
**Ascorbic Acid**	-	-	96 ± 0.9	-

No: No action under the used experimental conditions; ^a^ Values are means ± SD of three or four different determinations. Means within each column differ significantly (*p* < 0.05).

Several assays should be used in order to assess *in vitro* antioxidant activity. In this way, factors such as solubility or steric hindrance which may be of overriding importance in one environment but not in another can be varied. The used methods are associated with the generation of various radicals. Two types of approach have been taken under consideration: (i) the assays in which we have the scavenging by hydrogen- or electron donation of a preformed free radical as a marker of antioxidant activity as well as assays (ii) involving the presence of antioxidant system during the generation of the radical.

**Table 4:** Anticancer activity of cinnamic acids **1**–**4**. IC_50_ refers to the concentration of the compounds (in μM) required for 50% growth inhibition of human cancer cells of several types. MRC-5 refers to normal cells.

Compd.	IC_50_ HT-29 (μM)	IC_50_ A-549 (μM)	IC_50_ OAW-42 (μM)	IC_50_ MDA-MB-231 (μM)	IC_50_ HeLa (μM)	IC_50_ MRC-5 (μM)
**1i**	>240	>240	>240	>240	>240	222 ± 1.3
**1ii**	>>240	>>240	>>240	>>240	>>240	>>240
**2i**	141 ± 1.8	134 ± 1.6	160 ± 1.1	199 ± 1.9	117.5 ± 1.4	96.3 ± 1.1
**2ii**	>>240	>>240	>>240	>>240	240 ± 1.6	240 ± 1.0
**3i**	>240	>240	>240	>240	>240	>240
**3ii**	>>240	>>240	>>240	>>240	>>240	240 ± 1.8
**4i**	>240	210 ± 1.0	174 ± 1.8	250 ± 1.2	80 ± 0.9	92 ± 1.3
**4ii**	54 ± 1.1	173.5 ± 1.4	63.5 ± 0.8	47.5 ± 0.7	30 ± 0.4	24 ± 0.6

The novel acids were studied for the antioxidant activity by the use of the sTable 2,2-diphenyl-1-picrylhydrazyl radical (DPPH) at concentrations 0.05 and 0.1 mM after 20 and 60 min (Table 2) [23,24,25]. In the DPPH assay, the dominant chemical reaction involved is the reduction of the DPPH radical by single electron transfer SET from the antioxidant. Phenolic compounds eg. NDGA, giving phenoxide anions are effective antioxidants. The compounds presented reducing abilities which ranged nearly 29% (**4ii)**, small differences were found among the compounds with time and concentration. It has been found that phenyl-substituted acids present lower activity than the corresponding non-substituted (**2i** < **2ii**, **3i** < **3ii**, **4i** < **4ii**). Due to steric reasons the interaction of DPPH with the tested compounds is low.

Superoxide (O_2_^−**·**^) anion and hydroxyl radical (^.^OH) are free radical species of potential importance. OH reactive radical and ^1^O_2_ are responsible for cytotoxicity. During inflammation, ˙OH radicals are produced and are responsible for tissue damage.

Thus we evaluated our acids for their competition with DMSO for ˙OH radicals was measured, hydroxyl radicals were produced by the Fe^3+^/ascorbic acid system and expressed as percent inhibition of formaldehyde formation at 0.1 mM concentration (Table 3) [23,24,25]. Compounds **3i**, **3ii**, **4ii** presented high inhibitory activities of the oxidation of DMSO (33 mM) at 0.1 mM compared with the reference compound Trolox, while compound **2i** showed low inhibitory activity.

The superoxide anion radical (O_2_^−^·) is less toxic than the hydroxyl radical, but still being one of the most known toxic ROS. Two different experimental assays are followed for the determination of superoxide anion radical scavenging activity: (i) one involves an enzymatic production of superoxide anions and (ii) the other is supported by a non- enzymatic procedure. Herein the test is performed at 0.1 mM concentration, generating superoxide anion radicals by a non-enzymatically assay (Table 3). Compound **1i** presented the highest activity, followed by compound **1ii** (75% and 50%, respectively).

The ABTS^•+^ is derived from the oxidation of ABTS by potassium persulfate. It is a decolorization assay. The addition of electron-donating antioxidants leads to ABTS^•+^ reduction. The chemistry taking place involves the direct generation of the ABTS^•+^ with no involvement of an intermediary radical. The cation radical is formed prior to the addition of the antioxidant and does not take place continually in the presence of the antioxidant. All compounds presented moderate activity with the exception of compound **1ii** (47.4%) (Table 3). For the above mentioned test the results taken do not define the role of lipophilicity.

For the *in vitro* study of the free radical production, azo compounds generating free radicals through spontaneous thermal decomposition are used. The water soluble 2,2'-azobis(2-amidinopropane) hydrochloride (AAPH) is recommended as appropriate for measuring radical-scavenging activity *in vitro*. The activity of the peroxyl radicals produced by the action of AAPH greatly resembles to cellular activities such as lipid peroxidation. The phenyl substituted acids presented remarkable activity at 0.1 mM concentration (84%–92%) compared to the non-substituted derivatives (20%–63%). Compounds **1i**, **2i**, **3i**, **4i** with higher lipophilicity showed higher anti-lipid peroxidation response.

Epidemiological studies revealed the link between reactive oxygen species, inflammation and high cancer risk. In order to diminish the pro-cancerous mechanisms a key treatment strategy is to reduce the free radical load and consequently prevent potential damage to cellular compounds such as DNA, proteins and lipids. Moreover, it has been found that LOX metabolism as well as arachidonic acid metabolites play an important role in tumor progression and survival [40] and are implicated in the etiology of mammary carcinogenesis [41]. Studies have demonstrated that levels of several eicosanoids are increased in breast cancer in comparison to benign breast tumours [42,43]. Thus, LOX inhibitors may have chemopreventive activity in lung carcinogenesis [44,45].

Thus, we conducted * in vitro* studies in two colon cancer cell lines (HT-29 and HCT-15) reported time and dose-dependent stimulation of cell proliferation by LTB_4_ and 12-HETE [46]. The synthesized acids have been tested in five different human tumour cell lines: HT-29 (colon), A-549 (lung), OAW-42 (ovarian), MDA-MB-231 (breast), HeLa (immortal cells from cervical cancer) and MRC-5 normal cells. On the basis of the *in vitro* testing results, most of these compounds with different substitution patterns exhibited significant anticancer activity as depicted in Table 4.

For the HT-29 (colon cancer) compound **4ii** presented remarkable anticancer activity, followed by compound **2i**. For the A-549 (lung) cell lines, compound **2i** showed very good activity, followed by compounds **4ii** and **4i**. For the OAW-42 (ovary cancer) compounds **4ii**, **2i** and **4ii** possessed the better inhibitory anticancer activity. For the MDA-MB-231 (breast) cells compound **4ii** presented remarkable anticancer activity, followed by compounds **2i** and **4ii**. For the HeLa cervical cancer cells compounds **4ii** and **4i** showed the higher activities, followed by compounds **2i** and **2ii**. For the rest of the compounds the derived results were not satisfactory (>240 or >>240 μM ).

Normal lung MRC-5 cells found to be more resistant against the tested compounds (higher IC_50_ values). In all the cell lines 4-bromo-5-phenylpenta-2,4-dienoic acid (**4ii**) presents the best anticancer activity.

### 2.4. Computational Studies

#### 2.4.1. Computational Methods, Docking Simulations

All the molecules were constructed with the ChemDraw program [47] and converted into 3D-Structures with the OpenBabel program [48], by using MMFF94 force field. Protein setup was performed using the UCSF Chimera software [49,50]. Τhe AnteChamber PYthon Parser interfacE (ACPYPE) tool [51] was employed to generate the topologies of the ligands. ACPYPE tool is written in python to use Antechamber [52,53] to generate topologies for chemical compounds was used for the parameterization of the ligands. Energy minimizations where carried out with the molecular simulation toolkit GROMACS [54] using the AMBER99SB-ILDN force field [55].

Docking calculations were performed with the software Autodock Vina [56]. The PyRx program [57] was employed to generate the docking input files and to analyze the docking results. The proteins were considered rigid. Performing a blind docking the protein 1RRH soybean LOX, 4ii is properly aligned in the binding site of LOX. The single bonds were considered as active torsional bonds. An exhaustiveness value of 64 was used for the docking studies with a maximum output of 100 binding modes. The conformation results of ligands in the binding site of 1RRH are identical with the ones in 1IK3. The docking results, as a set of solutions, are ranked according to their scoring function values, defined by the 3D coordinates of its atoms and expressed as a PDB file.

#### 2.4.2. Molecular Docking Studies on Lipoxygenase

The lack of structural data for human LOX, lead us to model human LOX using soybean enzyme because of its availability and its well characterized structure [58]. For the docking studies we selected **4ii** which presents a good combination of *in vitro* anti-LOX result. The possible mechanism of action, as well as the differences in activity toward soybean LOX compared to the other derivatives could be explained by docking calculations.

For the docking studies of LOX we have used the 1RRH (soybean lipoxygenase) accessible from the Protein Data Bank (PDB) presenting a resolution of 2 Å [59]. We used the 1RRH from PDB with Fe^+3^ running blind docking. Two soybean LOX models were derived from 1RRH. The first one has the ligand taken from 1IK3 (ligand ID: 9OH) into the catalytic site while the other one for the blind docking simulation has no ligand. The metal center in both models has charge q = +3 with no bond restraint between the iron and the ligands. Lennard-Jones parameters for Fe (III) force field can be resumed as: σ_vdw_ = 2.138157e-01 nm, ε_vdw_ = 2.092e-1 kJ/mol. The confirmation of the docking study was accomplished with the crystallisation of the ligands in the protein of complex 1RRH. The results are in accordance with the structure of the molecules in the active site of the protein.

**Figure 1:**
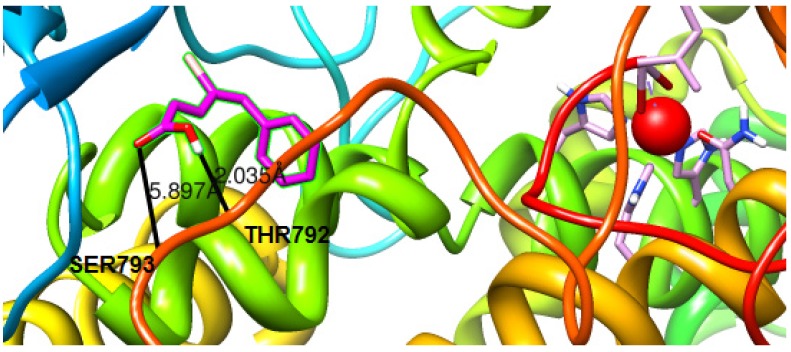
Docked poses of conjugate **4ii** (Magenta) in the LOX binding site. Ball and stick models are used to render the side-chains of relevant binding site residues. The iron ion is depicted as an orange sphere.

**Figure 2:**
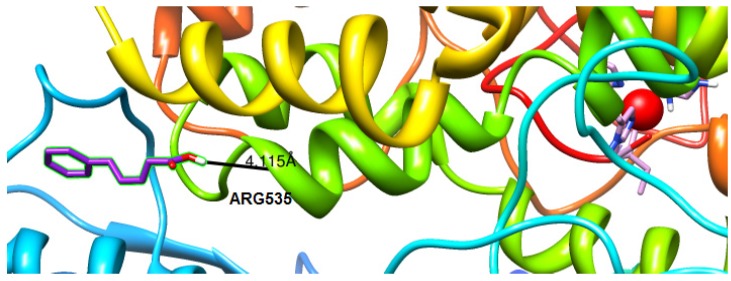
Docked poses of conjugate 3ii (Purple) in the LOX binding site. Ball and stick models are used to render the side-chains of relevant binding site residues. The iron ion is depicted as an orange sphere.

The docking study was performed for all the compounds. or the visualization of the docking results two compounds have been selected, **4ii** presenting the best anti-LOX activity and **3ii**, the simplest one of the series, presenting the lowest activity. The docking orientations of compound **4ii** are given in Figure 1 and of compound **3ii** in Figure 2. Compound **4ii** presents significant high binding energy to the protein (−6.8 kcal/mol) while compound **3ii** presents (−6.2 kcal/mol). A number of H-bonds were developed. These H-bonds were observed for compound **4ii** between: (a) oxygen of the carbonyl group with THR792 and (b) the oxygen of the hydroxyl group with SER793. For compound **3ii** H-bonds were developed between the oxygen of the carbonyl group with ARG535. Weak hydrophobic interactions were also observed, supporting an even stronger binding of the compounds to the LOX cavity.

Comparing the G-score of the series to the *in vitro* results it is concluded that the docking cannot really predict the *in vitro* activity under the reported experimental conditions, however it gives an idea for the interactions between the compounds and the active site.

## 3. Experimental

### 3.1. Materials and Instruments

All chemicals, solvents, chemical and biochemical reagents were of analytical grade and purchased from commercial sources. Soybean lipoxygenase, linoleic acid sodium salt, arachidonic acid (AA) were obtained from Sigma Chemical, Co. (St. Louis, MO, USA), 1,1-diphenyl-2-picrylhydrazyl (DPPH), nordihydroguairetic acid (NDGA) are purchased from the Aldrich Chemical Co. (Milwaukee, WI, USA).

Melting points (uncorrected) were determined on a MEL-Temp II (Lab. Devices, Holliston, MA, USA). UV-Vis spectra were obtained on a Perkin-Elmer 554 double beam spectrophotometer and on a Hitachi U-2001 spectrophotometer. Infrared spectra (film as Nujol mulls) were recorded with Perkin-Elmer 597 spectrophotometer (Perkin-Elmer Corporation Ltd., Lane Beaconsfield, Bucks, England) and a Shimadzu FTIR-8101M. The ^1^H-Nucleic Magnetic Resonance (NMR) spectra were recorded at 300 MHz on a Bruker AM 300 spectrometer (Bruker Analytische Messtechnik GmbH, Rheinstetten, Germany) in CDCl_3_ or DMSO using tetramethylsilane as an internal standard unless otherwise stated. ^13^C-NMR spectra were obtained at 75.5 MHz on a Bruker AM 300 spectrometer in CDCl_3_ or DMSO solutions with tetramethylsilane as internal reference unless otherwise stated. Chemical shifts are expressed in δ (ppm) and coupling constants *J* in Hz. Elemental analyses for C and H gave values acceptably close to the theoretical values (±0.4%) in a Perkin-Elmer 240B CHN analyzer. Reactions were monitored by thin-layer chromatography (TLC), on aluminum cards precoated with 0.2 mm of silica gel and fluorescent indicator.

### 3.2. Chemistry General Procedure

#### 3.2.1. Synthesis of Phenyl Substituted Cinnamic Acids 1i-4i [23,24,25]

Title compounds were prepared by a Knoevenangel reaction, as shown in Scheme 1. according to literature methods [23,24,25]. A suitable aldehyde (0.015 mol) was condensed with phenylacetic acid (0.015 mol) and acrylic acid anhydride (10 mL) in the presence of triethylamine (5 mL). The reaction mixture was refluxed for 5 h. The solution was poured into 2 N HCl, and ice and the formed precipitate was collected by filtration and recrystallized from 50% aqueous ethanol. In case that no precipitate was formed an extraction with 3 × 100 mL CHCl_3_ was made and the organic phase was collected, dried over MgSO_4_ and evaporated to dryness affording a residue that was recrystallized from 50% aqueous ethanol.

*3-(4-Cyanophenyl)-2-phenylacrylic acid* (**1i**) [30]. ^1^H-NMR (CDCl_3_): δ, (ppm) 7.39–7.46 (m, 7H), 7.51 (d, 1H, *J* = *9 Hz*), 7.56 (s, 1H), 7.65 (d, 1H, *J* = *9 Hz*) Anal. C, H, N. Calcd %: (C_16_H_11_NO_2_) C: 77.10, H: 4.45, N: 5.62 Found %: C: 76.96, H: 4.39, N: 5.68.

*5-(4-Nitrophenyl)-2-phenylpenta-2,4-dienoic acid* (**2i**). IR (Nujol) (cm^−^^1^): 3050-2940,1720, 1640 ^1^H-NMR (CDCl_3_): δ, (ppm) 6.95–7.06 (m, 1H), 7.31–7.33 (m, 2H), 7.45–7.50 (m, 7H), 7.73 (d, 1H, *J* = 9 Hz, phenyl), 8.16 (d, 1H, *J* = 9 Hz, Ph), 10.6 (s, 1H, COO*H*). ^13^C-NMR (CDCl_3_): 124.1 127.7, 128.2, 128.4, 128.7, 128.9, 129.9, 131.4, 134.2, 136.6, 140.1, 153.6, 163.4 Anal. C, H, N. Calcd %: (C_17_H_13_NO_4_) C: 69.15, H: 4.44, N: 4.74, Found %: C: 69.2, H: 4.61, N: 4.35.

*2,5-Diphenylpenta-2,4-dienoic acid* (**3i**) [31,32]. ^1^H-NMR (CDCl_3_): δ, (ppm) 7.33–7.41 (m, 11H), 7.82 (d, 1H, *J* = 9 Hz, Ph), 7.94 (d, 1H, *J* = 9 Hz, Ph). Anal. C, H. Calcd %: (C_17_H_14_O_2_) C: 81.58, H: 5.64, Found %: C: 81.46, H: 5.25.

4-Bromo-2,5-diphenylpenta-2,4-dienoic acid (**4i**). IR (Nujol) (cm^−^^1^): 3000-2900, 1750, 1630, 1480. ^1^H-NMR (CDCl_3_): δ, (ppm) 7.34–7.45 (m, 7H), 7.61–7.62 (b, 1H), 7.81–7.84 (m, 2H), 7.93–7.96 (m, 2H). ^13^C-NMR (CDCl_3_): 117.3, 126.2, 126.4, 127.8, 128.7, 128.8, 129.2, 129.9, 132.3, 133.3, 135.3, 137.2, 165.4. Anal. C, H. Calcd %: (C_17_H_13_BrO_2_) C: 62.03, H: 3.98, Found %: C: 62.05, H: 3.08.

#### 3.2.2. General Procedure of the Synthesis of Cinnamic Acids **1ii**–**4ii** [23,24,25]

The synthesis of the title compounds (Scheme 1) follows a Knoevenangel condensation of the suitable heteroarylaldehyde (0.01 mol) with malonic acid (0.01 mol) dissolved in 1.12 mL of pyridine and piperidine (0.01 mol). The mixture was refluxed until the emission of CO_2_ stopped. The reaction solution was poured into 2N HCl and ice and the formed precipitate was collected by filtration and recrystallized from water or from 3:1 water/ethanol. In the case that no precipitate was formed the water phase was extracted with 3 × 100 mL CHCl_3_ or CH_2_Cl_2_ and the organic phase was collected, dried over Mg_2_SO_4_, and evaporated to dryness affording a residue that was recrystallized from aqueous ethanol.

*3-(4-Cyanophenyl)acrylic acid* (**1ii**) [27]. ^1^H-NMR (DMSO): δ, (ppm) 6.53 (d, 1H, *J* = 15 Hz, CH*=*CH), 7.09 (d, 1H, *J* = 6 Hz), 7.43 (d, 1H, *J* = 6 Hz), 7.57–7.61 (s, 2H), 7.66 (d, 1H, *J* = 15 Hz, CH=CH). Anal. C, H, N. Calcd %: (C_10_H_7_NO_2_) C: 69.36, H: 4.07, N: 8.09, Found %: C: 69.49, H: 4.23, N: 8.00.

*5-(4-Nitrophenyl)penta-2,4-dienoic acid* (**2ii**) [28]. ^1^H-NMR (DMSO): δ, (ppm) 6.09 (d, 1H, *J* = 12 Hz, CH=CH=CH=CH), 6.93–7.14 (m, 2H, CH=CH=CH=CH), 7.42 (d, 1H, *J* =12 Hz, CH=CH=CH=CH), 7.66–8.21 (m, 4H, Ph). Anal. C, H, N. Calcd %: (C_11_H_9_NO_4_) C: 60.27, H: 4.14, N: 6.39, Found %: C: 59.95, H: 4.53, N: 6.79.

*5-Phenylpenta-2,4-dienoic acid* (**3ii**) [29]. ^1^H-NMR (CDCl_3_): δ, (ppm) 6.00 (d, 1H, *J* = 15 Hz, CH=CH), 6.86–6.99 (m, 1H), 732–7.40 (m, 4H), 7.47–7.50 (m, 3H). Anal. C, H. Calcd %: (C_11_H_10_O_2_) C: 75.84, H: 5.79, Found %: C: 76.08, H: 5.42.

4-Bromo-5-phenylpenta-2,4-dienoic acid (**4ii**). IR (Nujol) (cm^−^^1^): 3020–2900, 1750, 1650. ^1^H-NMR (CDCl_3_): 7.34–7.66 (m, 4H), 7.81–7.90 (m, 2H), 7.99–8.02 (m, 2H), 9.33 (s, 1H). ^13^C-NMR (CDCl_3_): 117.2, 118.3, 127.9, 128.1, 128.6, 136.8, 143.8, 170.4. Anal. C, H, N. Calcd %: (C_11_H_9_BrO_2_) C: 52.20, H: 3.58 Found %: C: 52.35, H: 3.62.

### 3.3. Physicochemical Studies

#### 3.3.1. Determination of R_M_ Values

Reversed phase TLC (RPTLC) was studied using silica gel plates saturated with 55% (*v/v*) liquid paraffin in light petroleum ether and methanol/water mixture (77/23, *v/v*) containing 0.1% of acetic acid was used as a mobile phase. Spots were detected under UV light or by iodine vapours. R_M_ values were determined by the corresponding R_f_ values (from ten individual measurements) using the equation R_M_ = log [(1/R_f_) − 1] [23,24,25].

#### 3.3.2. Estimation of Lipophilicity as Clog *P*

Bioloom of Biobyte Corp was used for the theoretical calculation of lipophilicity as Clog *P* values [34].

### 3.4. Biological Experiments

#### 3.4.1. Experiments *in vitro*

The *in vitro* assays were repeated at least in triplicate and the standard deviation of absorbance was less than 10% of the mean.

#### 3.4.1.1. Soybean Lipoxygenase Inhibition Study *in vitro* [23,24,25]

*In vitro* inhibitory LOX assay is accomplished as described previously. The novel derivatives for testing (stock solutions 10 mM in DMSO) were incubated at room temperature with sodium linoleate (0.1 mM) and 0.2 mL of LOX enzyme solution (1/9 × 10^−4^*w/v* in saline). The conversion of sodium linoleate to 13-hydroperoxylinoleic acid was measured at 234 nm and compared with the reference inhibitor. Several concentrations were used for the IC_50_ determination (Table 2).

#### 3.4.1.2. Interaction of the New Acrylic Acids with the Stable Radical 1,1-diphenyl-picrylhydrazyl (DPPH) [23,24,25]

To a solution of DPPH in absolute ethanol the appropriate volume of the compounds (0.05 and 0.1 mM final concentrations) dissolved in DMSO was added. The absorbance was recorded at 517 nm after 20 and 60 min at room temperature (Table 3).

#### 3.4.1.3. Hydroxyl Radicals Scavenging Activity [23,24,25]

The hydroxyl radicals were produced by the Fe ^3+^/ascorbic acid system. EDTA (0.1 mM), Fe ^3+^ (167 μM), DMSO (33 mM) in phosphate buffer (50 mM, pH 7.4), the tested compounds (0.1 mM) and ascorbic acid (10 mM) were mixed in test tubes. The solutions were incubated at 37 °C for 30 min. The reaction was stopped by CCl_3_COOH (17% *w/v*) (Table 4) and the % scavenging activity of the tested compounds for hydroxyl radicals was given.

#### 3.4.1.4. Superoxide Radical Scavenging Activity [23,24,25]

Superoxide radicals were produced non-enzymatically by mixing PMS, NADH and air–oxygen. The nitroblue tetrazolium method was used for the estimation of the produced radicals. The reaction mixture containing compounds (0.1 mM), 3 μM PMS, 78 μM NADH, and 25 μM NBT in 19 μM phosphate buffer pH 7.4 was incubated for 2 min at room temperature. The acrylic acids were preincubated for 2 min before adding NADH (Table 4) and the absorption was recorded at 560 nm.

#### 3.4.1.5. Inhibition of Linoleic Acid Peroxidation [25]

For initiating the free radical, 2,2'-azobis(2-amidinopropane) dihydrochloride (AAPH) is used. The final solution in the UV cuvette consisted of ten microliters of the 16 mM linoleate sodium dispersion 0.93 mL of 0.05 M phosphate buffer, pH 7.4, thermostatted at 37 °C. 50 μL of 40 mM AAPH solution was added as a free radical initiator at 37 °C under air and 10 μL of the tested compounds. The oxidation of linoleic acid sodium salt results a conjugated diene hydroperoxide. The reaction is monitored at 234 nm (Table 3).

#### 3.4.1.6. ABTS^+^-Decolorization Assay [25]

In this assay, ethanol is used for the dilution of the ABTS^+•^ solution. ABTS is dissolved in water to a 7 mM and reacts with 2.45 mM potassium phosphate to produce the ABTS radical cation (ABTS^+•^) by allowing the mixture to stand in the dark at room temperature for 12–16 h before use. Ten μL of diluted ABTS^+•^ solution (734 nm) is added to 10 μL of antioxidant compounds or Trolox standards (final concentration 0–0.1 mM) in ethanol and the absorbance is measured at 30 °C exactly 1 min after the initial mixing (Table 3). 

#### 3.4.1.7. Cytotoxic Activity

##### *Cell Lines* *and Culture Maintenance*

Human cancer cell lines used as targets were HT-29 (colon), A-549 (lung), OAW-42 (ovarian), MDA-MB-231 (breast), HeLa (immortal cells from cervical cancer). All cells were obtained from the American Type Culture Collection (ATCC). Cells were routinely grown as monolayer cell cultures in T-75 flasks (Costar) in an atmosphere containing 5% CO_2_ in air and 100% relative humidity at 37 °C and subcultured twice a week, restricting the total number of cell passages below 20. The culture medium used was Dulbecco’s modified Eagle’s medium (DMEM, Gibco, Grand Island, NY, USA) [60] supplemented with 10% fetal bovine serum (Gibco), 2 mM glutamine (Sigma), 100 μg/mL streptomycin and 100 IU/mL penicillin.

##### *Trypan* *Blue Exclusion*

The loss of membrane integrity, as a morphological characteristic for cell death, was assayed by Trypan Blue exclusion [61]. The number of cells that were alive was estimated through a haematocytometer and phase-contrast microscopy. Each result represented the mean of four independent measurements and used for the inoculation of cells in the microplates.

##### *Cell* *Inoculation–Drug Exposure–SRB Cytotoxicity Assay*

Cell passages were carried out by detaching adherent, logarithmically growing cells after addition of 2–3 mL of a mixture of 0.05% solution of trypsin (Gibco, 1:250) in phosphate-buffered saline (PBS) with 0.02% EDTA and incubation for 3–5 min at 37 °C. For the experiments, cells were plated (100 μL containing 10,000 cells/well) in 96-well flat-bottom microplates microplates (Costar-Corning, Acton, MA 01720, USA) [62] so that untreated cells were in exponential growth phase at the time of cytotoxicity evaluation. Cells were left for 24 h at 37 °C to resume exponential growth and stabilization and afterwards exposed to tested agents for 48 h by the addition of an equal volume (100 μL) of either complete culture medium (control wells), or twice the final drug concentrations diluted in complete culture medium (test wells). Drug cytotoxicity was measured by means of the SRB colorimetric assay estimating the survival fractions (SF) as the percent of control (untreated cells) absorbance. The SRB assay was carried out as previously described [63] and modified by our group [64]. In brief, culture medium was aspirated prior to fixation using a microplate-multiwash device (Tri-Continent Scientific, Inc., Grass Valley, CA, USA) and 50 μL of 10% cold (4 °C) trichloroacetic acid (TCA) were gently added to the wells. Microplates were left for 30 min at 4 °C, washed five times with deionized water and left to dry at room temperature for at least 24 h. Subsequently, 70 μL of 0.4% (*w/v*) sulforhodamine B (Sigma) in 1% acetic acid solution were added to each well and left at room temperature for 20 min. SRB was removed and the plates were washed five times with 1% acetic acid before air drying. Bound SRB was solubilized with 200 μL of 10 mM unbuffered Tris-base solution and plates were left on a plate shaker for at least 10 min. Absorbance was read in a 96-well plate reader at 492 nm subtracting the background measurement at 620 nm. The test optical density (OD) value was defined as the absorbance of each individual well, minus the blank value (“blank” is the mean optical density of the background control wells, *n* = 8). Mean values and the coefficient of variation (CV) from six replicate wells were calculated automatically. Results were expressed as the “survival fraction” (SF), as shown below. 

##### Calculation of Results

For each tested compound a dose-effect curve was produced. Sextuplicate determinations gave a CV (Standard Deviation/mean %) of much less than 10%, resulting in standard error (SE) which was very low in all cases. The data showing inhibition of cellular growth are expressed as the fraction of cells that remains unaffected (fu) (survival fraction, SF), which is derived from the following equation:

fu = ODx/ODc

where ODx and ODc represent the test and the control optical density, respectively. Drug potency was expressed in terms of IC_50_ values (50% inhibitory concentration) calculated from the plotted dose-effect curves (through least-square regression analysis) (Table 4).

## 4. Conclusions

The present study shows that the synthesized cinnamic acids constitute a promising class of antioxidant anticancer compounds. Docking studies support the *in vitro* anti-LOX activity indicating the entrance of **4ii** into the cavity of LOX. Compound **4ii** presented a promising anti-cancer activity, high LOX inhibitory activity as well as hydroxyl scavenging high and anti-LPO activities. Thus, acid **4ii** might be used as a lead compound for the design of new multifunction agents.

## Abbreviations

AA: Arachidonic Acid
AAPH: 2,2'-azobis (2-amidinopropane) hydrochloride
ACPYPE: AnteChamber PYthon Parser interface
clog *P*: Theoretically calculated lipophilicity
COX: Cyclooxygenase
DPPH: 2,2-Diphenyl-1-picrylhydrazyl radical
LDL: Low-density lipoprotein
LOX: Lipoxygenase
NBT: Nitroblue tetrazolium
ND: Neurodegenerative diseases
NDGA: Nordihydroguaretic acid
^•^OH: Hydroxyl radical
O_2_^−**·**^: Superoxide radical
ROS: Reactive oxygen species
RPTLC: Reverse-phase thin layer chromatography
TCA: Trichloroacetic acid
